# Clinical comparison of coronally-advanced flap plus amniotic membrane or subepithelial connective tissue in the treatment of Miller’s class I and II gingival recessions: A split-mouth study

**DOI:** 10.15171/joddd.2016.026

**Published:** 2016-08-17

**Authors:** Ardeshir Lafzi, Nader Abolfazli, Masoumeh Faramarzi, Masoumeh Eyvazi, Amir Eskandari, Fariba Salehsaber

**Affiliations:** ^1^Professor, Department of Periodontics, Faculty of Dentistry, Shahid Beheshti University of Medical Sciences, Tehran, Iran; ^2^Dental and Periodontal Research Center, Tabriz University of Medical Sciences, Tabriz, Iran; ^3^Associate Professor, Department of Periodontics, Faculty of Dentistry, Tabriz University of Medical Sciences, Tabriz, Iran; ^4^Assistant Professor, Department of Periodontics, Faculty of Dentistry, Kermanshah University of Medical Sciences, Kermanshah, Iran; ^5^Assistant Professor, Department of Periodontics, Faculty of Dentistry, Tabriz University of Medical Sciences, Tabriz, Iran; ^6^Assistant Professor, Department of Periodontics, Faculty of Dentistry, Tabriz University of Medical Sciences, Tabriz, Iran

**Keywords:** Amniotic membrane, gingival recession, connective tissue

## Abstract

***Background.*** The aim of the present study was to compare coronally advanced flap (CAF) plus amniotic membrane (AM) to CAF with connective tissue graft (CTG) in the treatment of Miller’s class I and II gingival recessions.

***Methods.*** Eleven healthy subjects with thirty Miller’s class І and ІІ gingival recessions ≥3 mm, were selevted for this research and randomly assigned to two groups in a split-mouth design. In the control group gingival recessions were treated with CAF and CTG; however, in the test group the lesions were treated with (AM) and CAF. The clinical parameters, including recession depth (RD), recession width (RW), keratinized tissue width (WKT), probing depth (PD) and clinical attachment level (CAL), were measured at baseline and 1, 3 and 6 months postoperatively. Statistical significance was set at P < 0.01.

***Results.*** Position changes of RD, RW, CAL, and MGJ were significant between baseline and one month after surgery (P < 0.01) in both the test and control groups and these values remained unchanged at 3- and 6-month follow-ups. There were no statistically significant differences in PD and WKT between baseline and 1-, 3- and 6-months intervals postoperatively. The mean root coverage values after 6 months were 75.5% and 63.1% for two groups, respectively. The mean recession depth reductions were 2.63±0.63 mm and 2±1.4 mm in the test and control groups, respectively.

***Conclusion.*** The results of this research showed that application of AM instead of connective tissue decreased surgical operation time and patient discomfort but the amount of root coverage was not significantly different between the two methods.

## Introduction

Gingival recession can be seen in people with both poor and good oral hygiene.^1-3^The incidence of gingival recession varies from 8% in children to 100% after 50 years of age.^4-5^Several aspects of gingival recession such as esthetic/cosmetic demands and root sensitivity make it clinically significant, requiring surgical correction. Another common indication for the treatment of root coverage procedures is to level off the marginal soft tissue with the aim of better plaque control.^5^ Pedicle grafts,^7^ free grafts,^8^ guided tissue regeneration,^9^ and subepithelial connective tissue grafts (SCTG)^10,11^ are the most commonly used surgical procedures in the treatment of root exposures.

SCTG technique is considered as the “gold standard” of root coverage procedures.^10^Although this method has high predictability and results in successful root coverage in the long term, its source is limited and significantly leads to patient discomfort.^12^ If we use an alternative source for the donor site, surgical stages will be reduced and the morbidity of patient following root coverage surgery would decrease.^13^

**Figure 1.**
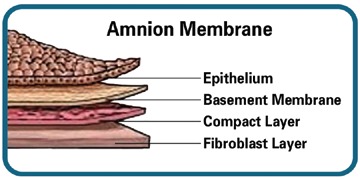
Histological structure of human amniotic membrane.

Amniotic membrane (AM) is an allograft that is derived from human amniotic tissue. It is the innermost layer of fetal membranes and has a thin epithelial layer, a thick basement membrane and an avascular stroma consisting mainly of collagen (Figure 1). Studies have found that amniotic membrane stimulates re-epithelialization, decreases inflammatory response and modulates angiogenesis. It has shown antibacterial properties and low immunogenicity.^14-18^Also, studies have demonstrated that AM is rich in some growth factors like basic fibroblast growth factor (b FGF), epidermal growth factor (EGF), transforming growth factor-α (TGF-α), transforming growth factor-β (TGF-β), hepatocyte growth factor (HGF) and keratinocyte growth factor (KGF).^17-19^

The usefulness of AM has been reported in the treatment of many medical problems.^20-23^ In dentistry Kothiwale et al^24^ used AM as guided tissue regeneration (GTR) for the management of human periodontal Grade II buccal furcation defects with two different bone substitute materials. Reduction of CAL, PD, bone fill and percentage gain in furcation area was significant in this treatment.^24^ Valez et al used amniotic membrane in implant surgery and showed that AM is effective in helping cicatrization and wound healing.^25^Ghahrodi et al used AM for root coverage treatments. The results of a study showed that connective tissue graft might be successfully replaced by connective tissue graft in surgeries for root coverage.^26^

Based on the biologic properties of amniotic membrane and its potential, we theorized that it would be effective in the management of root exposure. The aim of the present study was to compare the clinical effectiveness of amniotic membrane and coronally advanced flap to subepithelial connective tissue with a coronally advanced flap in Miller class І and II root exposures.

## Methods

The study was approved by the research Ethics Committee of Tabriz University of Medical Sciences (5/4/9997) and registered with the local World Health Organization Registry Network (IRCT138808142670N1).

### Study population 

In this split-mouth, blind, randomized clinical trial eleven subjects with a mean age of 34 ± 12 years were selected. Thirty defects were randomly divided into two groups: control group (treated with CAF + SCTG) and test group (treated with CAF+AM) by flip of a coin. All the patients met the inclusion and exclusion criteria. Inclusion criteria included Miller class I and II buccal recessions with minimum 3 mm of RD on the premolars, canines and incisors in both the left and right quadrants of the same jaw in the mandible or maxilla. The width of keratinized gingiva measuring ≥2 mm^25^was a prerequisite for accomplishing the CPF. All the patients met the inclusion. Exclusion criteria consisted of systemic conditions affecting the periodontium, bleeding on probing in surgical sites, poor oral hygiene (plaque index ≥20%), pregnancy, steroid therapy, history of root canal therapy, history of root coverage surgery, symptoms of pulpal pain in selected teeth, PDL widening, periapical radiolucency, need for antibiotic prophylaxis, orthodontic therapy, high frenal and muscular attachments and smoking and teeth with buccal and lingual inclinations. The selected teeth included 4 central incisors, 4 lateral incisors, 12 canines, 8 first premolars and 2 second premolars. A total of 20 defects were in the maxilla and 10 defects in the mandible.

### Pre-surgical procedures

The study procedures were explained to all the participants. The protocol of this clinical trial was approved by the Ethics Committee and Research Vice-chancellor of Tabriz University of Medical Sciences under the code IRCT138808142670N1. All the patients signed an informed consent form.

Phase I periodontal treatment was carried out, including thorough scaling and root planing (SRP) with ultrasonic scalers and hand instruments for all the patients. Four to six weeks after initial phase of the treatment, a reassessment was made to evaluate PD, CAL, BOP and mobility. For all the participants, an O´Leary plaque score^28^<20% and GI^29^ ≤1 was prerequisite before undertaking surgical phase of the therapy. Impressions were taken from the affected areas and acrylic stents with guiding grooves were fabricated to attain reproducible measurements. A standard UNC periodontal probe was used for measurements. All the parameters were measured at baseline and at 1-, 3- and 6-month postoperative interevals: recession width (RW), plaque index (PI), probing depth (PD), gingival index (GI), recession depth (RD), clinical attachment level (CAL), and width of keratinized tissue (WKT). RD was recorded from cemento-enamel junction (CEJ) to the gingival margin. RW was recorded as a line that connected the two points at the most coronal parts of the recession. A single examiner blinded to the study design and grouping recorded all the clinical measurements. Root coverage percentage was determined 1, 3 and 6 months after surgery.

### Surgical procedure

**Figure 2.**
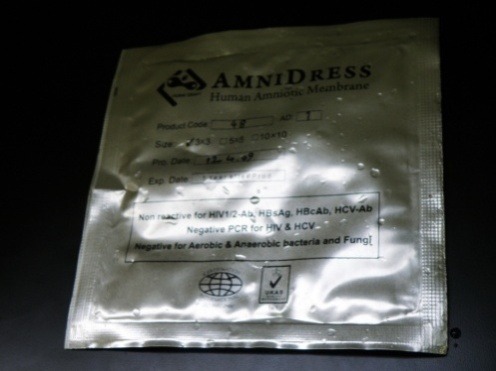
AmniDress®.

**Figure 3.**
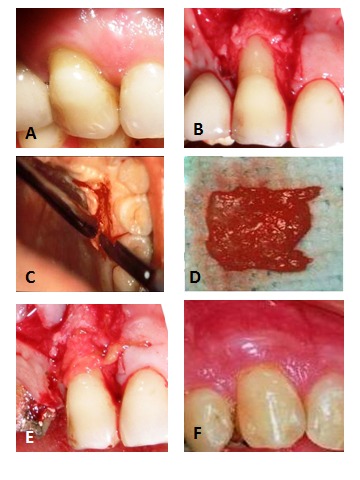
Control group: A) Recession defect on the right maxillary canine; B) Preparation of the recipient site; C & D) Harvesting the connective tissue graft; E) The connective tissue graft was placed over the exposed root surface; F) Complete root coverage at 6-month follow-up.

**Figure 4.**
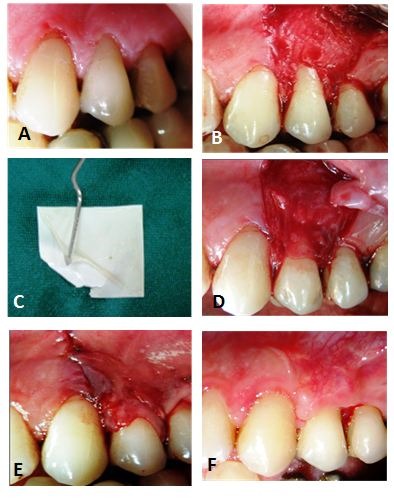
Test group: A) Recession defect on the vleft maxillary bicuspid; B) Preparation of the recipient site; C) Preparation of the amniotic membrane; D) The amniotic membrane was placed over the exposed root surface; E) The flap was positioned coronally over the amniotic membrane and secured with a sling sutures; F) Partial root coverage at 6-month follow-up.

One surgeon carried out all the surgical procedures with local anesthesia (2% lidocaine with 1:80000 epinephrine). Each subject received two horizontal incisions at the level of the CEJ followed by two oblique vertical incisions extending beyond the mucogingival junction (MGJ) and a partial-thickness flap was reflected. The surface epithelium of the adjacent interdental papilla was removed. In the control group, the CTG was prepared with a palate origin and sutured in the target site.^9^Then the partial-thickness flap was secured in a coronal position with a sling suture technique to cover the connective tissue graft (Figure 2). In the test group the human amniotic membrane, (AmniDress®, ImenChemiNarin Ltd Co, Tehran, Iran.) (Figure 3) was removed from cryo-box container approximately 5‒10 minutes prior to surgery according to the manufacture^’^s protocol. The cryo-preserved AM was trimmed for the recipient site size and transferred to the recipient site. Upon placement, the amniotic membrane adhered to the recipient root and proximal site, thus eliminating the need to suturing. The prepared flap was positioned coronally over the amniotic membrane and sutured in place with a sling technique. The surgical areas were covered with periodontal dressing (Figure 4).

### Post-surgical considerations

All the patients were instructed to use chlorhexidine mouthrinse (0.2%) (Iran Najo, Tehran, Iran) twice a day for two weeks. To prevent postsurgical infection 500-mg amoxicillin capsules (Alhavi, Tehran, Iran) were prescribed three times a day for a week. Oral hygiene instructions included discontinuing tooth brushing near the surgical site. Sutures and periodontal dressing were removed 14 days after surgery. All the participants were seen two, four, twelve, and twenty-four weeks following surgery and in each session, the grafted defect sites were re-measured with a standard periodontal probe. Mechanical therapy was carried out for the patients during the study.

### Statistical analysis

The parameters were reported as means ± standard divisions (SD). Inter-group differences were compared before and after the treatment, using ANOVA, and t-test was used to compare the results between the two groups at baseline and 1, 3, and 6 months postoperatively. P < 0.01 was considered statistically significant.

## Results

Thirty Miller’s class I and II root exposures in eleven patients were selected for this study. The average changes for the study variables from baseline to the sixth month in the control and test groups are shown in Table 1. There were no statistically significant differences between the control and test groups at the baseline (P > 0.01). In both groups, improvement of PD and WKT from baseline to 1, 3 and 6 months were not statistically significant. The changes of RW, RD, CAL and the CEJ-MGJ distance were significant between baseline and first month after surgery, remaining stable during future follow-ups. The initial mean recession depth (RD) changed from 3.43 ‏± 0.63 mm to 0.8 ‏± 0.8 mm and 3.13‏±0.4 mm to 1.13 ‏± 1.26 mm (P < 0.01) for the control and test groups, respectively. There were no statistically significant differences between the two groups in RW, RD, CAL and the CEJ-MGJ distance (P > 0.01). However, root coverage percentage was higher in the control group compared to the test group (75.5% vs. 63.1%) and the differences were not statistically significant (P = 0.331). Complete root coverage was detected in 66.6% (10 defects) of the subjects in the control group compared to 46.6% (7 defects) of the subjects in the test group 6 months after surgery.

**Table 1:** The measured variables (mean ± SD) in the test and control groups at baseline and1, 3, and 6 months after surgery

		**Baseline**	**1 month**	**3 month**	**6 month**
**PD (mm)**	**Control**	1±0.33	1.16±0.36	0.9±0.34	0.86±0.3
	**Test**	1.17±0.56	1.17±0.31	1.07±0.26	1.03±0.3
**RW (mm)**	**Control**	4.5±0.5	2.2±1.07^*^	2.1±1.04^*^	2.1±1.04
	**Test**	4.33±0.84	1.73±1.55^*^	1.7±1.54^*^	1.66±1.67
**RD (mm)**	**Control**	3.43±0.63	0.83±0.82*	0.76±0.73*	0.8±0.8
	**Test**	3.13±0.4	1.2±1.13^*^	1.13±1.13^*^	1.13±1.26
**CAL (mm)**	**Control**	4.43±0.9	1.99±0.96^*^	1.66±0.78^*^	1.66±0.86
	**Test**	4.3±0.62	2.37±1.19^*^	2.2±1.13^*^	2.16±1.31
**WKT (mm)**	**Control**	3.53±1.2	3.3±1.07	3.3±0.71	3.53±0.83
	**Test**	3.13±0.3	3.07±0.18	3.2±0.32	3.23±0.32
**CEJ-MGJ (mm)**	**Control**	7±1.16	4.33±1.05^*^	4.4±0.87^*^	4.4±0.87
	**Test**	6.26±0.5	4.26±1.16	4.33±1.06	4.36±1.13
**ROOT COVERAGE (%)**	**Control**		74.49±26	6.09±7.24	75.54±26.2
	**Test**		61.32±36.4	63.55±36.2	63.18±40.6

PD: probing depth, RW: recession width, RD: recession width, CAL: clinical attachment level, WKT: width of keratinized tissue, CEJ-MGJ: cemento-enamel junction to muco-gingival junction ^*^Indicates statistical significance.

## Discussion

The aim of this study was to compare the use of AM for the CTG, together with a CAF, in the management of Miller Class I and II root exposures. Both treatment procedures reduced RD, RW and CAL at 6 months significantly. There were no statistically significant changes in clinical parameters between the test and control groups, although the control group achieved clinically superior results.

The recorded GI and PI in the test and control group at baseline were ≤20% and ≤1, respectively, remaining stable during future follow-ups. A similar result was obtained in relation to PD, consistent with other studies.^30-32^The enhancement of CAL from baseline to the first month after surgery was statistically significant in the study groups. No significant changes were found between the first month and 3 or 6 months after surgery. The CAL gain in the control and test groups averaged 2.76 ± 0.86 mm and 2.1 ± 1.31 mm, respectively. Other researchers reported CAL gain ranging from 2.3 mm to 5.1 mm, with an insignificant change in probing depth, following the use of CAF + CTG.^30-32^ Nevertheless, determination of the nature of the resultant attachment without histological evaluation is not possible. The WKT did not increase in the control and test groups, although MGJ moved coronally. There are a few studies available on changes in WKT after root coverage surgery. It has been shown that WKT may decrease slightly 5‒12 months after coronally advanced flap surgery.^33-34^ But long-term studies have indicated that gingival dimensions may increase.^35-36^Chambrone in a Cochrane systematic review included clinical trials with a study duration of ≥6 months and found that subepithelial connective tissue graft procedures significantly increased WKT.^34^Due to the short duration of this study, more prolonged follow-ups are suggested.

In this study, the RD reduction was 0.33 mm more in the control group compared to the test group (P = 0.39). RD decreased from 3.43 ± 0.63 mm to 0.8 ± 0.8 mm and 3.13 ± 1.4 mm to 1.13 ± 1.26 mm in the control and test group, respectively. Harris et al^30^ reported superior results in RD reduction (3.68 mm 3 months after surgery) using SCTG and CAF. However, in Harris study, the baseline RD was less than that in this study. Huang et al showed that the amount of recession depth changes in coronally advanced flap technique is positively related to the baseline recession depth.^37^

Average of RW reduction was 2.4 ± 1.04 mm in the control group and 2.66 ± 1.82 mm in the test group, respectively. A reduction of 2.7 ± 1.2 mm in RW after 6 months was found in Wang et al^38^ research.

Harris et al^30^demonstrated that recession width decreased from 3.5± 1 mm to 0.7 ± 1.2 mm and from 3.6 ± 1.2 mm to 0.4 ± 1 mm after three months and one year, respectively. The mean root coverage was 63.18% and 75.54% in the control and test groups after 6 months, respectively, with no significant differences between the two groups. The mean root coverage in the control group is consistent with the results of previous studies, indicating a mean root coverage of 86% (53‒98%).^39^This discrepancy may partly relate to differences in study designs, research duration, follow-up intervals and statistical analyses. Ghahroudi et al^26^ in a research similar to our study compared the efficacy of AM and CTG in the management of root exposure. Average root coverage rates after 6 months in the two groups were 67% and 54%, respectively. The mean percentage of RC in our study was greater than that in this study; this difference might be attributed to differences in the design of the present study. The present study design was split-mouth, which could reduce the effects of confounding factors.

Two important factors in the success of surgical root coverage include bridging and creeping attachment.^27^Borghetti and Gardella^40^pointed out that any improvement in the coverage of exposed root 1 month after surgery can be related to the creeping attachment. In the present study, we found 0.23 mm and 0.16 mm of creeping attachment in two groups between the first and the sixth months. Two prognostic factors affecting the success of coronally advanced flap (CAF) are tension flap and flap thickness.^41^In this study, after coronal placement of the flap, the extension of the vertical incision limited flap tension, and the flap was fixed with interrupted sutures in its new position.

## Conclusion

Obviously, SCTG with CAF is the gold standard for the treatment of Miller class I and II gingival recession defects, and amniotic membrane with CAF may be relatively comparable with gold standard. This new allograft decreases the duration of surgery and morbidity of the patient. In fact, satisfaction with esthetic results of amniotic membrane is higher.

## Acknowledgments

The authors would like to acknowledge the Dental and Periodontal Research Center of Tabriz University of Medical Sciences for their support in the development and realization of the present study.

## Authors’ contributions

AL, NA, MF, ME were responsible for the main design and concept. AE and FS performed the literature search. AL and MF contributed in data acquisition and analysis in this research. MF and NA drafted the manuscript. All the authors have read and approved the final manuscript.

## Funding

The work was supported by the Dental and Periodontal Research Center, Tabriz University of Medical Sciences, Tabriz, Iran.

## Competing interests

The authors declare that they have no competing interests with regards to authorship and/or publication of this article.

## Ethics approval

The study was approved by the Research Ethics Committee of Tabriz University of Medical Sciences (5/4/9997) and registered with the local World Health Organization Registry Network (IRCT138808142670N1).
